# Quantitative proteomics of extracellular vesicles derived from human primary and metastatic colorectal cancer cells

**DOI:** 10.3402/jev.v1i0.18704

**Published:** 2012-09-11

**Authors:** Dong-Sic Choi, Do-Young Choi, Bok Sil Hong, Su Chul Jang, Dae-Kyum Kim, Jaewook Lee, Yoon-Keun Kim, Kwang Pyo Kim, Yong Song Gho

**Affiliations:** 1 Department of Life Science and Division of Molecular and Life Sciences, Pohang University of Science and Technology, Pohang, Republic of Korea; 2 Department of Molecular Biotechnology, Konkuk University, Seoul, Republic of Korea

**Keywords:** colorectal cancer, microvesicles, exosomes, ectosomes, metastasis, biomarker, secretome, APEX, label-free quantitative proteomics, nanoparticle tracking analysis

## Abstract

Cancer cells actively release extracellular vesicles (EVs), including exosomes and microvesicles, into surrounding tissues. These EVs play pleiotropic roles in cancer progression and metastasis, including invasion, angiogenesis, and immune modulation. However, the proteomic differences between primary and metastatic cancer cell-derived EVs remain unclear. Here, we conducted comparative proteomic analysis between EVs derived from human primary colorectal cancer cells (SW480) and their metastatic derivatives (SW620). Using label-free quantitation, we identified 803 and 787 proteins in SW480 EVs and SW620 EVs, respectively. Based on comparison between the estimated abundance of EV proteins, we identified 368 SW480 EV-enriched and 359 SW620 EV-enriched proteins. SW480 EV-enriched proteins played a role in cell adhesion, but SW620 EV-enriched proteins were associated with cancer progression and functioned as diagnostic indicators of metastatic cancer; they were overexpressed in metastatic colorectal cancer and played roles in multidrug resistance. As the first proteomic analysis comparing primary and metastatic cancer-derived EVs, this study increases our understanding of the pathological function of EVs in the metastatic process and provides useful biomarkers for cancer metastasis.

Mammalian cells release extracellular vesicles (EVs) with diameters ranging from 50 nm to 1 µm into their environment (1). EVs are spherical lipid bilayer structures that contain proteins, genetic materials and bioactive lipids (1–3). Two mechanisms of EV biogenesis have been proposed. First, exosomes, with diameters of 50–100 nm, are secreted from the endosomal membrane compartment after the fusion of multivesicular bodies with the plasma membrane (1). Second, microvesicles bud directly from the plasma membrane with diameters ranging from 100 nm to 1 µm (1). However, numerous similarities exist with respect to the physical characteristics and compositions of exosomes and microvesicles (4,5), resulting that exclusive purification of the target vesicles after their secretion from cells has been challenging (4). Therefore, we collectively refer to these vesicles as EVs (5).

Cancer cell-derived EVs play a role in immune escape, neovascularization and metastasis (3,6–9). In addition, mRNA transcripts related to the cell cycle were found to modulate the proliferation of endothelial cells in CRC-derived EVs (10). However, a comparison between the proteomes of primary and metastatic cancer cell-derived EVs has not been performed. Thus, we investigated EVs derived from 2 isogenic CRC cells from the same patient: the primary cancer cells (SW480; Dukes’ type B) and their lymph node metastatic derivatives (SW620; Dukes’ type C). These 2 types of CRC cells with the same genetic background but different metastatic potentials have been previously used as models for CRC metastasis studies (11,12).

Using a combination of OFFGEL fractionation of in-solution digested peptides, liquid chromatography–tandem mass spectroscopy (LC-MS/MS) analyses and label-free quantitation via the absolute protein expression (APEX) tool (13), we identified that SW480 EV-enriched proteins played a role in cell adhesion and represented the cellular status of cells. In contrast, SW620 EV-enriched proteins were associated with cancer progression and functioned as diagnostic indicators of metastatic cancer with poor prognosis. Our study is the first to use high-throughput proteomic analysis to compare primary and metastatic cancer-derived EVs to increase our understanding of the pathological function of EVs in the metastatic process and provide useful biomarkers for cancer metastasis.

## Materials and methods

### Cell cultures

The primary cell line SW480 and its lymph node metastatic cell line SW620 from the same patient with CRC were cultured in RPMI-1640 (Invitrogen Corporation, Carlsbad, CA, USA) supplemented with 10% heat-inactivated foetal bovine serum (Invitrogen Corporation), 100 U/mL penicillin and 0.1 mg/mL streptomycin at 37°C in 5% (v/v) CO_2_ air atmosphere.

### Purification of EVs

EV purification was performed as previously described (10). Briefly, SW480 cells (~5.1×10^9^) and SW620 cells (~11.4×10^9^) at 80–90% confluency were washed twice with phosphate-buffered saline (PBS) and then incubated in serum-free RPMI-1640 for 24 h. Approximately 2,000 mL of conditioned medium was collected, centrifuged at 500*g* for 10 min and then centrifuged twice at 2,000*g* for 15 min. We concentrated the supernatant using a QuixStand Benchtop System with a 100 kDa hollow fiber membrane (GE Healthcare, Bucks, UK), and then added the concentrate (~36 mL) to both ultracentrifuge tubes which contain 1.0 mL of 0.8 M and 0.5 mL of 2.0 M sucrose cushions in buffer A (20 mM HEPES, 150 mM NaCl, pH 7.4). After ultracentrifugation at 100,000*g* for 2 h, 2 mL of the interface between the 0.8 and 2.0 M sucrose cushions was collected together from both ultracentrifuge tubes and diluted 5-fold in PBS. The sample was added to 0.35 mL of 0.8 M and 0.15 mL of 2.0 M sucrose cushions, and centrifuged at 100,000*g* for 2 h. The EVs (0.5 mL) were harvested from the interface between the 0.8 and 2.0 M sucrose cushions, diluted with 1.8 mL of buffer A and mixed with an equal volume of 60% iodixanol solution (Axis-Shield PoC AS, Oslo, Norway). This sample was placed at the bottom of a tube and overlaid with 3 mL of 20% and 2.5 mL of 5% iodixanol. After centrifugation at 200,000*g* for 2 h, 10 fractions of equal volume (1 mL) were collected from the top of the gradient. The concentration of EV proteins was quantified using the Bradford assay. From the culture supernatants of ~5.1×10^9^ SW480 cells and ~11.4×10^9^ SW620 cells, we obtained 123.2 and 125.0 µg of EVs in total proteins, respectively.

### Western blotting

Whole cell lysates and EVs were separated by SDS-PAGE and then transferred to a polyvinylidene difluoride membrane. The membrane was blocked, incubated with primary antibodies followed by horseradish peroxidase-conjugated goat anti-mouse IgG (Santa Cruz Biotechnology, Santa Cruz, CA, USA) or donkey anti-goat IgG (Santa Cruz Biotechnology) and subjected to enhanced chemiluminescence. Goat anti-ACTB, mouse anti-CD63, mouse anti-CTNNB1, mouse anti-LAMP1 and mouse anti-PARP antibodies were purchased from Santa Cruz Biotechnology. Mouse anti-CD9 and anti-CD81 antibodies were from BD Biosciences (San Diego, CA, USA). Goat anti-EGFR and anti-ICAM1 antibodies were obtained from R&D Systems (Abingdon, UK), and mouse anti-PDCD6IP was from Cell Signaling Technology (Beverly, MA, USA).

### Nanoparticle tracking analysis

The diameters of the purified EVs (500 ng/mL in PBS) were measured using a NanoSight LM10 system equipped with a 405 nm laser (NanoSight Ltd., Amesbury, UK) at 25°C (14). Samples were introduced manually and the video images were recorded for 30 sec using the nanoparticle tracking analysis (NTA) software (version 2.3): blur setting, auto; camera level, 9; detection threshold, 5; minimum expected particle size, auto; minimum track length, 10; viscosity, 0.89 cP. Each experiment was carried out in triplicate. The NanoSight was calibrated with 100 and 200 nm polystyrene beads.

### Electron microscopy

The purified EVs were applied to glow-discharged carbon-coated copper grids (EMS, Matfield, PA, USA). After allowing the EVs to absorb for 3 min, the grids were rinsed with droplets of deionised water and positive-stained with a mixture of 2% methylcellulose and 4% uranylacetate (Ted Pella, Redding, CA, USA). Electron micrographs were recorded using a JEM 1011 microscope (Jeol, Tokyo, Japan) at an acceleration voltage of 100 kV.

### In-solution digestion and peptide OFFGEL fractionation

Purified EVs (100 µg) were delipidated by methanol/chloroform precipitation. Precipitated pellet was resolved in a digestion solution of 6 M urea and 50 mM ammonium bicarbonate in HPLC-grade water. Protein reduction was performed with 5 mM Tris (2-carboxyethyl) phosphine hydrochloride (Thermo Scientific, Rockford, IL, USA) for 1 h, followed by an alkylation step with 25 mM iodoacetamide (Sigma-Aldrich, St Louis, MO, USA) in the dark for 30 min at room temperature. The sample was digested in-solution with 2 µg (enzyme to protein ratio 1:50) of sequencing-grade modified trypsin (Promega, Madison, WI, USA) for 16 h at 37°C and desalted with the Sep-Pak column (Waters, Milford, MA, USA). The digested peptides were fractionated using an Agilent 3100 OFFGEL fractionator system (Agilent, Santa Clara, CA, USA). The 24-cm immobilised IPG gel strip with linear pH gradient 3–10 (Agilent) was rehydrated with 40 µL/well IPG-rehydration buffer for 15 min. The peptide was dissolved in 2.8 mL off-gel stock solution and 150 µL of sample was loaded into each well. The peptides were focused with 50 µA and 8,000 V for approximately 40 h. After separation, 2 adjacent wells were combined and desalted with a PepClean C18 spin column (Thermo Scientific) for MS analysis.

### Nano-LC-ESI-MS/MS

The purified tryptic peptides were analysed using an LTQ-Orbitrap mass spectrometer (Thermo Finnigan, San Jose, CA, USA) coupled with Eksigent-Nano-Ultra (Eksigent Technologies, Dublin, CA, USA). Tryptic peptides were applied to a home-made analytic column (75 µm×12 cm) packed with C18 regular 5-µm sized resin. A linear 60 min gradient was used from 97% solvent A (0.1% formic acid in H_2_O) to 60% solvent B (0.1% formic acid in ACN) at a flow rate of 0.3 µL/min. The separated peptides were electrosprayed into the nanoESI source. The electrospray voltage was 1.9 kV using 35% normalised collision energy for MS/MS. All MS/MS spectra were acquired in data-dependent scans for fragmentation of the 5 most abundant spectrums from full MS scans. The repeat count for dynamic exclusion was set to 1, the repeat duration was 30 s, the dynamic exclusion duration was set at 180 s, the exclusion mass width was 1.5 Da and the list of dynamic exclusion was 50.

### Identification and quantification of proteins

We performed peptide identification from LC-MS/MS data of 12 fractions with 2 technical replicates using X!!Tandem (version Dec-01-2008) in the computational proteomics analysis system (CPAS; version 9.2) (15). Each raw data file was first converted to mzXML using the Trans-Proteomic Pipeline (TPP; version 4.3) (16). MS/MS scans in the converted mzXMLs were then subjected to a search against the UniProt human database (release 2011_02) using X!!Tandem. The tolerance was set to 10 ppm for precursor ions and 1 Da for fragment ions. Enzyme specificity for trypsin was used. The oxidation of methionine (15.995 Da), carbamidomethylation of cysteine (57.021 Da), deamination of N-terminal glutamine (−17.027 Da) and dehydration of N-terminal glutamic acid (−18.011 Da) were selected as a variable modification. The number of missed cleavage sites was set to 2. We finally used TPP to statistically identify peptides and proteins from the X!!Tandem search results by PeptideProphet (p≥0.9) and ProteinProphet (p≥0.95), respectively. All proteomic data sets are deposited in the PRIDE database (http://www.ebi.ac.uk/pride) under the accession numbers 21768 (SW480 EVs) and 21769 (SW620 EVs).

To measure the relative quantity of each protein in our EV proteomes, we calculated relative protein abundance using the APEX tool (13). The first 100 top-ranked proteins with high spectral counts from ProteinProphet results were selected as a training data set to generate an experiment-specific data set. With an APEX normalization factor of 100,000, the protein abundance was then calculated as described in Lu et al. (13).

### Gene Ontology annotation

For Gene Ontology (GO) analysis, gene IDs or protein accession numbers were converted to Swiss-Prot accession numbers. Protein lists were imported into the DAVID Bioinformatics database and assigned to their GO terms as a cellular component, biological process or molecular function (17).

## Results and discussions

### Quantitative proteomic analysis of SW480 EVs and SW620 EVs

**Fig. 1:**
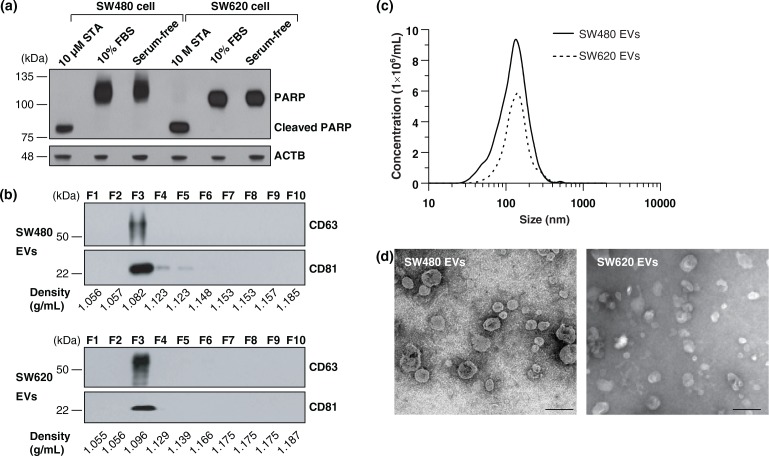
Purification and characterization of SW480 EVs and SW620 EVs. (a) Cells were incubated with media containing 10% FBS or serum-free media for 24 h. Serum-free medium did not induce apoptosis under our experimental conditions, as shown by an assay for cleaved PARP. As a positive control for apoptosis, cells were treated with 10 µM of staurosporine (STA) in serum-free media for 24 h (68). (b) Fractions of OptiPrep density gradients were analysed by Western blotting. CD63 and CD81, marker proteins of EVs (1), were detected in fraction 3. (c) The size distribution of EVs was measured by NTA indicating an average diameter of 159.1±11.1 nm (ranged from 23.0 to 636.3 nm) and 165.5±8.6 nm (ranged from 26.7 to 574.7 nm) for SW480 EVs and SW620 EVs, respectively. (d) Electron microscopy revealed that purified EVs were small closed vesicles lacking apoptotic bodies, cellular debris or protein aggregates. Scale bar = 200 nm.

EVs were purified from the cell culture supernatant incubated with serum-free medium to avoid potential contamination from serum-derived vesicles and proteins (5,8,18,19). An assay for cleaved PARP indicated that apoptosis was not induced by incubation of SW480 and SW620 cells in serum-free medium for 24 h (Fig. 1a). A combination of differential centrifugation, ultrafiltration using a 100 kDa hollow fiber membrane, ultracentrifugation onto sucrose cushions and OptiPrep density gradient ultracentrifugation was conducted (10). The EVs derived from SW480 and SW620 settled at a similar density of ~1.082 and ~1.096 g/mL in the OptiPrep density gradient fractions, respectively, as indicated by the EV marker proteins CD63 and CD81 (Fig. 1b) (10). NTA showed that the average diameters of the SW480 EVs and SW620 EVs were 159.1±11.1 nm (ranged from 23.0 to 636.3 nm) and 165.5±8.6 nm (ranged from 26.7 to 574.7 nm), respectively (Fig. 1c). An examination of the purified EVs using electron microscopy revealed that they were small closed vesicles lacking apoptotic bodies, cellular debris or protein aggregates (Fig. 1d).

**Fig. 2:**
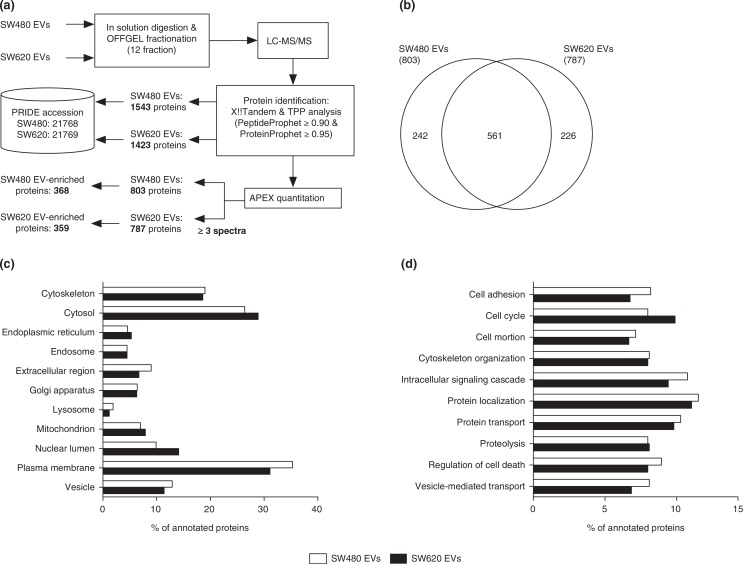
Quantitative proteomic analysis of SW480 EVs and SW620 EVs. (a) Schematic workflow of the quantitative proteomics of EVs indicates the number of proteins in each step. (b) The Venn diagram shows the overlapped proteins in SW480 EVs and SW620 EVs. The GO analyses for cellular components (c) and biological processes (d) of SW480 EVs and SW620 EVs. Note that proteins normally have several GO annotations.

Proteins of purified EVs were in-solution digested, separated with OFFGEL electrophoresis and analysed by LC-MS/MS (Fig. 2a). We identified a total of 1,543 and 1,423 proteins from SW480 EVs and SW620 EVs, respectively (PeptideProphet≥0.9 and ProteinProphet ≥0.95, estimated false discovery rate; 0.006 in SW480 EVs and 0.005 in SW620 EVs) (Table S1). The abundance of each EV protein was estimated by the APEX tool based on spectral counting of each protein and corrected by the prior expectation of observing each peptide (13). To improve the confidence of protein quantification, we only analysed proteins with more than 3 spectra. In total, 803 and 787 proteins were identified in SW480 EVs and SW620 EVs, respectively (Fig. 2b and Table S2).

To examine the overall proteomic composition of SW480 EVs and SW620 EVs, we categorised the identified EV proteins using GO annotation. The GO analyses showed that the cellular component and biological process annotations of SW480 EVs and SW620 EVs were similar (Fig. 2c and 2d). Both EV proteomes are composed of diverse proteins localised to the plasma membrane, cytosol and cytoskeleton rather than the mitochondrion, endoplasmic reticulum and other subcellular compartments (Fig. 2c). Biological process annotations revealed that proteins of the SW480 EVs and SW620 EVs played a role in cell adhesion, cell cycle, cell motion, cytoskeleton organization, intracellular signalling cascade, protein localization, protein transport, proteolysis, regulation of cell death and vesicle-mediated transport (Fig. 2d). Overall, these proteomic compositions of SW480 EVs and SW620 EVs are consistent with those of previously reported CRC EV proteomes (8,19,20).

### Comparison between EV proteomes and secretomes of SW480 and SW620 cell

**Fig. 3:**
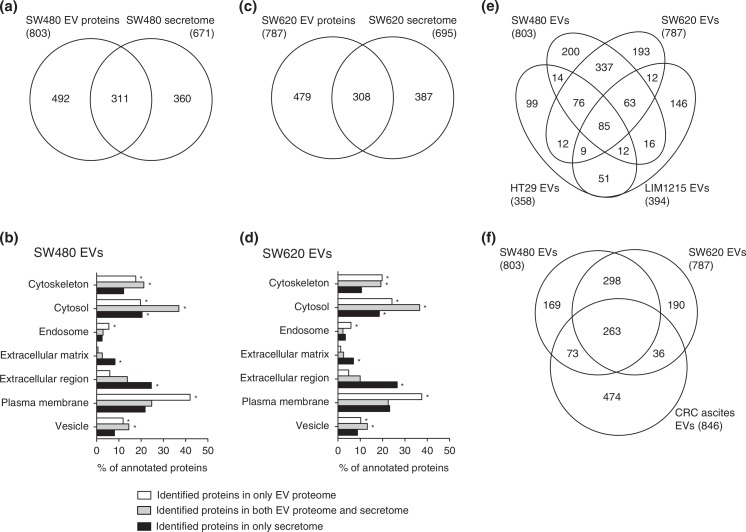
Comparison of EV proteomes with secretomes and other CRC cell-derived EV proteomes. The number of identified proteins and GO cellular component annotation were compared between EVs and secretomes (11) derived from SW480 (a and b) and SW620 (c and d). Note that proteins normally have several GO annotations. p<10^−5^ is denoted by *. The Venn diagrams show the overlap of proteins identified by SW480 EVs and SW620 EVs with HT29 EVs and LIM1215 EVs (e) and CRC ascites-derived EVs (f).

We next compared our EV proteomes with the previously reported secretomes of SW480 and SW620 cells (Fig. 3a–3d) (11). We found that 311 (38.7%) SW480 vesicular proteins overlapped with the SW480 secretome and identified 308 proteins in both EV proteome and secretome of SW620 cells (Fig. 3a and 3c). Several of these overlapped proteins originated from the cytosol, cytoskeleton and plasma membrane (Fig. 3b and 3d). Many of these proteins were common vesicular proteins such as 14-3-3 proteins, annexins, CD9 and MSN (Table S2).

Conditioned medium used for secretome analysis is mainly composed of secreted proteins and shed plasma membrane proteins, while plasma membrane, cytoskeletal and endosomal proteins are highly enriched in EVs (19,21). The proteins found exclusively in the secretomes of SW480 and SW620 cells were significantly annotated with extracellular matrix and the extracellular region, as expected (Fig. 3b and 3d). In contrast, proteins localised to the cytoskeleton, endosome and plasma membrane were enriched in the EVs compared to the secretomes of cells (Fig. 3b and 3d). These vesicle-enriched cytoskeletal and endosomal proteins are involved in vesicle biogenesis, structure and trafficking (21). Moreover, vesicle-enriched plasma membrane proteins have diverse pathophysiological functions in EVs and are potential biomarkers of various diseases including cancer (22). Therefore, our results suggest that the selective enrichment of plasma membrane proteins in cancer cell-derived EVs minimises the detection of highly abundant soluble proteins and maximises the identification of disease-related surface proteins in serum (19,21). These results support that the proteomes of cancer-derived EVs can be used instead of secretomes as an alternative source of cancer biomarkers (21).

### Comparison of CRC-derived EV proteomes

EVs harbour cell-specific proteins as well as common vesicular proteins involved in the biogenesis and structure of EVs (20). High-throughput proteomic analyses of CRC cell-derived EVs have been performed in HT29 (8), LIM1215 (20) and ascites derived from patients with histologically proven stage IV CRC (19). Note that HT29 and LIM1215 are well or moderately differentiated in CRC cells, respectively, while SW480 and SW620 are poorly differentiated cells. In this study, we compared our EV proteomes with those of HT29-, LIM1215- and CRC ascites-derived EVs (Table S2).

As shown in Fig. 3e and Table S3, 85 proteins overlapped in SW480 EVs, SW620 EVs, HT29 EVs and LIM1215. Most of these proteins are common in the EV proteomes such as 14-3-3 proteins (YWHAB, YWHAE, YWHAG, YWHAH, YWHAQ and YWHAZ), annexin proteins (ANXA2, ANXA4 and ANXA11), cytoskeletal proteins (ACTB, ACTR3, CFL1, KRT1, KRT10, KRT8, KRT9, PFN1 and TUBB), heat shock proteins (HSP90AA1, HSPA8 and HSPD1), integrin proteins ITGA2, ITGA6, ITGAV, ITGB1 and ITGB4), metabolic proteins (AHCY, ENO1, FASN, GAPDH, GSTP1, PGK1, PKM2, PPIA and UGDH), motor proteins (DYNC1H1, MYH10, MYH9 and MYL6), small GTPase proteins (ARF3, IQGAP1, RAB10, RAB5C, RAB7A, RALA, RALB and RAN), transporter/channel proteins (ATP1A1, ATP5B, SLC12A2, SLC1A5, SLC2A1, SLC3A2 and SLC44A1), tetraspanin and their associated protein (CD9, CD81 and PTGFRN), vesicle trafficking-associated proteins (EHD4, PDCD6, PDCD6IP, SDCBP, VCP, VPS35 and VPS37B) and other EV marker proteins such as MFGE8 (23). Some overlapped proteins play a role in CRC progression such as ADAM10, CD44, MIF and TAGLN2 (8,24).

Next, we compared our EV proteomes with that of CRC ascites-derived EVs (Figure 3f). A total of 263 proteins overlapped, most of which are common in EV proteomes and are known to overlap with HT29 EVs and LIM1215 EVs. However, some of these overlapped proteins were not identified in HT29 EVs and LIM1215 EVs; these proteins play a role in cytoskeleton organization (ACTR2, ARPC1B, ARPC2, CAPZA2, CAPZB, CORO1B, CPNE3 and FSCN1) and cancer progression (CD276, EPCAM and ICAM1). Cytoskeleton networks regulate cancer development-related cellular events such as cell polarity, differentiation, proliferation, migration and invasion (25). In CRC, loss of cytoskeleton organization results in abnormal regulation of cell adhesion and junctions, inducing the dedifferentiation and invasive cancer phenotypes (25). Our results show that EVs derived from poorly differentiated CRC cells (SW480, SW620 and CRC ascites) harbour more diverse cytoskeleton proteins than those from more differentiated cells (HT29 and LIM1215). Although the relationship between the loss of cytoskeleton organization and sorting of cytoskeleton-related proteins into EVs remains unclear, loading of diverse cytoskeleton proteins on EVs may induce the less differentiated phenotype of cells, reflecting characteristics of the cellular origin.

### Comparative proteomic analysis of SW480 EVs and SW620 EVs

**Fig. 4:**
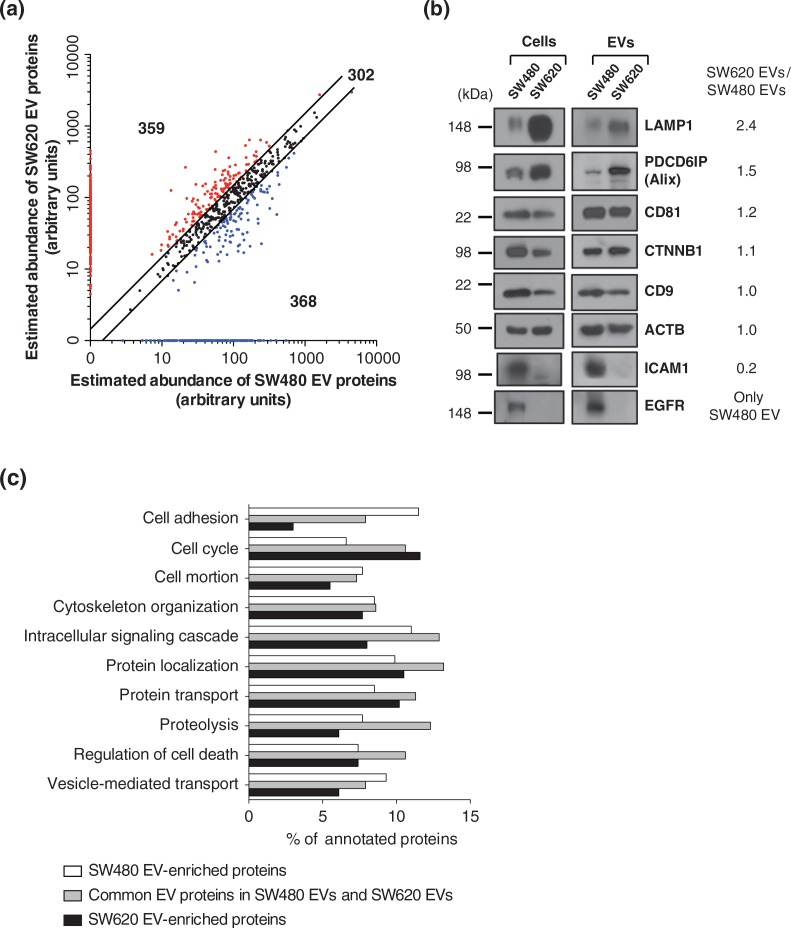
Comparative proteomic analysis of SW480 EVs and SW620 EVs. (a) Using the APEX tool (13), the relative abundances of vesicular proteins were calculated and their abundances were given in arbitrary units. Black dots show the 302 common EV proteins with expression changes of less than 1.5-fold. Blue dots indicate the 368 SW480 EV-enriched proteins with more than a 1.5-fold upregulation in protein abundance compared to SW620 EVs or only identified in SW480 EVs. Red dots denote the 359 SW620 EV-enriched proteins with more than a 1.5-fold upregulation in protein abundance over SW480 EVs or only identified in SW620 EVs. (b) Predicted fold-changes based on quantitative proteomics were validated by Western blotting of whole cell lysates (50 µg) and EVs (2 µg). Results are representative of 2 experiments. (c) Proteomes of common EV proteins, SW480 EV-enriched proteins and SW620 EV-enriched proteins were analysed by GO biological process annotations. Note that proteins normally have several GO annotations.

Based on the comparison of our EV proteomes with secretomes and other EV proteomes of CRC cells or ascites, we found that EVs are enriched in plasma membrane proteins and composed of proteins representing the cellular origin. We next compared the protein abundances between SW480 EVs and SW620 EVs. We categorised these proteomes into 3 categories (Table S4): 302 common EV proteins, which had less than a 1.5-fold change in protein abundance; 368 SW480 EV-enriched proteins, which were more than 1.5-fold upregulated compared to SW620 EVs or only identified in SW480 EVs; and 359 SW620 EV-enriched proteins, which were more than 1.5-fold upregulated compared to SW480 EVs or only identified in SW620 EVs (Fig. 4a). The reliability of our comparative proteomic data was examined using Western blotting of 8 EV proteins (Fig. 4b). LAMP1 and PDCD6IP (known as an Alix) are marker proteins of endosome-related intracellular vesicles (4). CD81 and CD9 are tetraspanins and well-known EV marker proteins (1). CTNNB1 is a frequently identified EV protein in CRC-derived EVs (8,19). ICAM1 and EGFR are also frequently identified in EVs (9,23). We found that Western blotting results corroborated the predicted fold-changes by the APEX tool (13). However, CD81 and CD9 did not exactly correlate with the abundance ratio. Note that the abundance ratio of these proteins was less than 1.5.

Next, we analysed these categorised proteins based on GO biological processes (Fig. 4c). Proteins that play a role in cytoskeleton organization, intracellular signalling cascades, protein localization, protein transport, proteolysis and regulation of cell death were mainly categorised as common EV proteins. In contrast, proteins involved in cell adhesion, cell motion and vesicle-mediated transport were upregulated in SW480 EVs while cell cycle-related proteins were upregulated in SW620 EVs.

### Cell adhesion-associated proteins enriched in SW480 EVs compared to SW620 EVs

Normal cells lose their cellular adhesion properties as they progress toward malignant cancers (26). Thus, cancer cell-derived EVs might represent the cell adhesion characteristics of the cellular origin. Our results show that cell adhesion-related proteins such as integrin-associated proteins, adhesion-related proteins and junction proteins were loaded in EVs and their abundances were differentially regulated in SW480 EVs and SW620 EVs (Table I). Most integrin-associated proteins were enriched in SW480 EVs including ILK, ITGA1, ITGA3, ITGA5, ITGAV, ITGB1, ITGB5 and LIMS1 (excluding ITGA6 and ITGB4). We also identified other adhesion-related proteins enriched in SW480 EVs compared to SW620 EVs such as PPFIBP1, L1CAM, ICAM1, PTK7, CD44, SYK, SCRIB, TGFBI, ERBB2IP, TPBG, CASK, STXBP3 and NME1. The abundance of these adhesion proteins in EVs seems to follow their cellular origin expression levels. A previous study reported that the expression of cellular adhesion and focal adhesion-related proteins in SW620 was downregulated compared to SW480 (12). Our result shows that the cellular level of ICAM1 was upregulated in SW480 compared to SW620, similar to the difference between SW480 EVs and SW620 EVs (Fig. 4b). Also, ITGA3, ITGB1 and CD44 are enriched in SW480 compared to SW620 (27,28). Taken together, our results and previous reports suggest that the abundance of adhesion-related proteins in EVs correlates with the cellular abundance.

Next, we evaluated the abundance of junction proteins in our EV proteomes. Previous proteomic analyses of cancer cell-derived EVs show that junction proteins are loaded in EVs (8,19). The sorting mechanism and potential roles in cancer progression remain unclear but the cellular expression of CTNNB1 is downregulated via EV-associated export from the cell (29), suggesting that EVs play a role in the elimination of cellular proteins from the cell. The loss of cellular junctions induces the invasive and metastatic potential of cancer cells. For example, adheren junctions and desmosomes are downregulated in epithelial cancer cells according to their malignancy (30). We found that various adherens junction-associated proteins such as CDH13, CTNNA1, CTNNB1, CTNND1, DLG1, JUP, MLLT4 and VCL were enriched in EVs and several desmosome-associated proteins including DSG2, PKP2, PKP3 and PKP4 were identified in our EV proteomes (Table I). Most of these adherens junction proteins were not differentially regulated between SW480 EVs and SW620 EVs, but desmosome-associated proteins including DSG2, PKP2 and PKP4 were enriched in SW480 EVs compared to SW620 EVs. Desmosomes reinforce the cellular adhesion and loss of desmosome-associated proteins promotes anchorage-independent cancer growth (30). Therefore, our results suggest that the abundance of desmosome-associated proteins in EVs is associated with the progression toward malignancy.

**Table I:** Cell adhesion-associated EV proteins enriched in SW480 EVs compared to SW620 EVs

Protein	UniProt accession number	Gene symbol	Estimated abundance of vesicular protein		Fold change (SW620 EVs/SW480 EVs)

			SW480 EVs	SW620 EVs	
Integrin-associated protein					
Calcium and integrin-binding protein 1	Q99828	CIB1		68.0	
Galectin-3-binding protein	Q08380	LGALS3BP		91.0	
Integrin alpha-1	P56199	ITGA1	14.7		
Integrin alpha-2	P17301	ITGA2	70.0	53.8	0.8
Integrin alpha-3	P26006	ITGA3	105.4	19.7	0.2
Integrin alpha-5	P08648	ITGA5	21.1		
Integrin alpha-6	P23229	ITGA6	223.4	179.9	0.8
Integrin alpha-V	P06756	ITGAV	163.4	15.9	0.1
Integrin beta-1	P05556	ITGB1	196.3	110.1	0.6
Integrin beta-4	P16144	ITGB4	210.7	144.9	0.7
Integrin beta-5	P18084	ITGB5	151.3	37.7	0.2
Integrin-linked protein kinase	Q13418	ILK	32.0		
Lactadherin	Q08431	MFGE8	335.0	271.8	0.8
LIM and senescent cell antigen-like-containing domain protein 1	P48059	LIMS1	90.0		
Vitronectin	P04004	VTN		32.4	
Other adhesion-related protein					
40S ribosomal protein SA	P08865	RPSA	66.4	141.7	2.1
ADP-ribosylation factor 6	P62330	ARF6	306.8	290.3	0.9
Alpha-parvin	Q9NVD7	PARVA	40.3		
Amyloid beta A4 protein	P05067	APP	26.1	24.6	0.9
Basement membrane-specific heparan sulfate proteoglycan core protein	P98160	HSPG2		7.9	
CD2-associated protein	Q9Y5K6	CD2AP	41.4	45.5	1.1
CD9 antigen	P21926	CD9	935.7	964.9	1.0
CD44 antigen	P16070	CD44	281.9	62.4	0.2
CD97 antigen	P48960	CD97	72.5	58.8	0.8
CD151 antigen	P48509	CD151	102.6	78.6	0.8
Cell surface glycoprotein MUC18	P43121	MCAM	126.7		
Coagulation factor V	P12259	F5	11.0		
Coronin-1A	P31146	CORO1A	27.6		
Discoidin, CUB and LCCL domain-containing protein 2	Q96PD2	DCBLD2	18.7	23.8	1.3
Disintegrin and metalloproteinase domain-containing protein 15	Q13444	ADAM15	31.6		
Ephrin-B1	P98172	EFNB1	151.7		
Epidermal growth factor receptor	P00533	EGFR	98.8		
Ezrin	P15311	EZR	243.4	166.8	0.7
Fermitin family homolog 2	Q96AC1	FERMT2	14.7		
Flotillin-2	Q14254	FLOT2	17.6	16.9	1.0
G-protein coupled receptor 56	Q9Y653	GPR56		85.0	
GDP-mannose 4,6 dehydratase	O60547	GMDS	28.8	60.2	2.1
Intercellular adhesion molecule 1	P05362	ICAM1	381.5	67.9	0.2
LIM domain only protein 7	Q8WWI1	LMO7		9.1	
Liprin-beta-1	Q86W92	PPFIBP1	91.5	14.5	0.2
Lymphocyte function-associated antigen 3	P19256	CD58	126.9		
Moesin	P26038	MSN	317.3	236.1	0.7
Myelin protein zero-like protein 2	O60487	MPZL2	75.4		
Myosin-9	P35579	MYH9	110.4	155.9	1.4
Neural cell adhesion molecule L1	P32004	L1CAM	69.9	11.8	0.2
Neuropilin-1	O14786	NRP1	22.6		
Neuroplastin	Q9Y639	NPTN	40.2	34.6	0.9
Nucleoside diphosphate kinase A	P15531	NME1	542.4	349.2	0.6
Peripheral plasma membrane protein CASK	O14936	CASK	20.4	11.2	0.5
Poliovirus receptor-related protein 2	Q92692	PVRL2	53.4		
Protein LAP2	Q96RT1	ERBB2IP	16.7	7.2	0.4
Protein scribble homolog	Q14160	SCRIB	57.9	18.7	0.3
Protein sidekick-1	Q7Z5N4	SDK1	6.7		
Rho-related GTP-binding protein RhoB	P62745	RHOB	44.2		
Sorbin and SH3 domain-containing protein 1	Q9BX66	SORBS1	22.1		
Syntaxin-binding protein 1	P61764	STXBP1	68.2		
Syntaxin-binding protein 3	O00186	STXBP3	105.6	62.7	0.6
Transforming growth factor-beta-induced protein ig-h3	Q15582	TGFBI	56.8	23.0	0.4
Transforming protein RhoA	P61586	RHOA	427.8	432.8	1.0
Trophoblast glycoprotein	Q13641	TPBG	228.0	110.0	0.5
Tyrosine-protein kinase SYK	P43405	SYK	66.7	20.8	0.3
Tyrosine-protein kinase transmembrane receptor ROR2	Q01974	ROR2	19.5		
Tyrosine-protein kinase-like 7	Q13308	PTK7	60.8	12.6	0.2
Junction protein					
Afadin	P55196	MLLT4	15.3	8.4	0.6
Cadherin-13	P55290	CDH13	33.8	37.5	1.1
Catenin alpha-1	P35221	CTNNA1	146.2	176.1	1.2
Catenin beta-1	P35222	CTNNB1	181.1	204.9	1.1
Catenin delta-1	O60716	CTNND1	144.9	153.9	1.1
Desmoglein-2	Q14126	DSG2		10.7	
Disks large homolog 1	Q12959	DLG1	157.9	82.7	0.5
Junction plakoglobin	P14923	JUP	78.6	66.3	0.8
Junctional adhesion molecule A	Q9Y624	F11R	183.0	131.1	0.7
Plakophilin-2	Q99959	PKP2	158.3	101.9	0.6
Plakophilin-3	Q9Y446	PKP3	84.2	89.4	1.1
Plakophilin-4	Q99569	PKP4	31.4	12.2	0.4
Vinculin	P18206	VCL	47.3	112.7	2.4
Zyxin	Q15942	ZYX		38.4	

### CRC progression- and metastasis-related proteins enriched in SW620 EVs compared to SW480 EVs

The proteins with the highest-fold change in SW620 EVs/SW480 EVs were AARS, CCDC50, VIM, NACA, DDX5, ANXA1, TCP1, HNRNPD, VPS37B and PA2G4 (Table S2). We found that many multivesicular bodies-associated proteins are enriched in SW620 EVs. For example, both endosomal sorting complex required for transport (ESCRT)-I proteins (FAM125A, TSG101, VPS28 and VPS37B) and ESCRT-III proteins (CHMP1B, CHMP2A, CHMP4A, CHMP4B, CHMP4C and CHMP5) are required for the sorting of ubiquitinated cargo into the intraluminal vesicles of multivesicular bodies (31). Also, PDCD6IP, which plays a targeting function in ESCRT complex (31), and VPS4A, which regulates ESCRT-III disassembly (31), were enriched in SW620 EVs. Collectively, upregulation of ESCRT proteins in SW620 EVs suggests that biogenesis of exosomes (32) may be more activated in the SW620 cells than in the SW480 cells. However, detailed mechanisms should be further analysed.

Interestingly, several SW620 EV-enriched proteins play a role in cancer progression and function as diagnostic indicators of metastatic cancer with poor prognosis; they are also overexpressed in CRC/metastatic cancer and associated with multidrug resistance (Table II). FSCN1 (33), MARCKS (34), PAK4 (35), MYO10 (36), MYO1B (37) and VCL (38) were overexpressed in metastatic cancer cells. These proteins are involved in cell shape, migration and invasion. In particular, FSCN1 is expressed at the invasive front of CRC cells facilitating their migration and invasion via remodelling of the actin cytoskeleton (33). Also, MARCKS may regulate the metastasis of cancer cells by modulating cell adhesion, secretion and motility through the actin cytoskeletal structure (34). Overexpression of CD276 in CRC cells is known to protect against cancer by inducing natural-killer cell death and is involved in cancer vasculature (39). PROM1, a pentaspan membrane glycoprotein, was initially described as a specific surface marker for human hematopoietic stem cells. Importantly, cancer-initiating cells within the prominin-1-positive population are able to maintain themselves, as well as differentiate and re-establish cancer heterogeneity (40). Also, PROM1-containing EVs are found in human cerebral fluids and appear to be upregulated in patients with glioblastoma, suggesting that it is associated with cancer progression. STRAP, upregulated in CRCs, enhances tumorigenicity by promoting anchorage-independent growth (41). Increased expression of YBX1, the transcription/translation regulatory protein, is associated with enhanced metastatic potential accompanying an epithelial-mesenchymal transition with poor patient survival (42). These observations suggest that EVs from metastatic cancer cells may function in metastasis.

**Table II:** CRC progression- and metastasis-related proteins enriched in SW620 EVs compared to SW480 EVs

Protein	UniProt accession number	Gene symbol	Estimated abundance of vesicular protein		Fold change (SW620 EVs/SW480 EVs)	SW480 secretome	SW620 secretome	HT29 EVs	LIM1215 EVs	CRC asictes EVs	Reference

			SW480 EVs	SW620 EVs							
Cancer progression											
CD276 antigen	Q5ZPR3	CD276	34.1	90.8	2.7					O	39
Fascin	Q16658	FSCN1	108.1	176.3	1.6	O	O			O	33
Myristoylated alanine-rich C-kinase substrate	P29966	MARCKS	144.9	298.9	2.1			O		O	34
Myosin-10	P35580	MYH10	31.2	73.6	2.4			O	O	O	36
Myosin-Ib	O43795	MYO1B	98.9	154.6	1.6					O	37
Serine/threonine-protein kinase PAK 4	O96013	PAK4		42.7							35
Prominin-1	O43490	PROM1		107.3						O	40
Serine-threonine kinase receptor-associated protein	Q9Y3F4	STRAP	43.0	78.3	1.8						41
Vinculin	P18206	VCL	47.3	112.7	2.4	O	O			O	38
Nuclease-sensitive element-binding protein 1	P67809	YBX1		88.9							42
Diagnostic indicator of metastatic cancer with poor prognosis											
Annexin A1	P04083	ANXA1	35.4	148.9	4.2		O	O		O	43
Annexin A11	P50995	ANXA11	86.6	161.8	1.9			O	O	O	43
High mobility group protein HMG-I/HMG-Y	P17096	HMGA		254.6							44
Overexpressed protein in CRC											
Dipeptidase 1	P16444	DPEP1		118.1					O		45
Formin-like protein 1	O95466	FMNL1		10.2							46,47
Formin-like protein 2	Q96PY5	FMNL2		18.1							46,47
Lysosome-associated membrane glycoprotein 1	P11279	LAMP1	40.8	96.2	2.4			O		O	48,49
Tyrosine-protein kinase Lck	P06239	LCK		289.1						O	50
Plastin-2	P13796	LCP1		166.9						O	51
Metastasis-associated in colon cancer protein 1	Q6ZN28	MACC1		28.0							52
Solute carrier family 12 member 2	P55011	SLC12A2	29.5	56.2	1.9			O	O	O	53,54
Transgelin-2	P37802	TAGLN2	189.0	436.6	2.3	O	O	O	O	O	24
Vimentin	P08670	VIM	36.9	177.8	4.8	O	O				55
Overexpressed protein in other metastatic cancer											
Adenylate kinase 2, mitochondrial	P54819	AK2		121.5							56
Stress-70 protein, mitochondrial	P38646	HSPA9		32.8				O			57
Galectin-3-binding protein	Q08380	LGALS3BP		91.0					O		58
Protein S100-A14	Q9HCY8	S100A14		177.9				O	O	O	61
Protein S100-A4	P26447	S100A4		250.1						O	59–61
Protein S100-A6	P06703	S100A6		139.8				O		O	59,60
Protein S100-A9	P06702	S100A9		90.6						O	59,60
Syntenin-1	O00560	SDCBP	435.7	755.0	1.7	O	O	O	O	O	62
Syntenin-2	Q9H190	SDCBP2		118.4					O	O	62
Multidrug resistance											
Multidrug resistance-associated protein 4	O15439	ABCC4	28.0	45.2	1.6						64
Calcyclin-binding protein	Q9HB71	CACYBP		158.1							12,66
Glutathione S-transferase P	P09211	GSTP1	295.9	596.3	2.0	O	O	O	O	O	67

ANXA1, ANXA11 and HMGA are overexpressed in metastatic cancer cells and are used as diagnostic indicators of poor prognosis. The annexins bind to negatively charged phospholipids in a calcium-dependent manner. ANXA1 and ANXA11 expression increases in various cancers including CRC with poor prognosis (43). HMGA, a non-histone chromatin protein that alters chromatin structure, is considered a poor prognostic indicator (44). Overexpression of HMGA correlates with the presence of metastasis and a low survival rate (44).

Several SW620 EV-enriched proteins such as DPEP1, FMNL1, FMNL2, LAMP1, LCK, LCP1, MACC1, SLC12A2, TAGLN2 and VIM are overexpressed in CRC and metastatic CRC cells. DPEP1, a zinc metalloprotease, is highly expressed in CRC and its expression is correlated with tumour aggressiveness and poor prognosis (45). FMNL1 and FMNL2 expression, which controls cell motility and invasion in an actin-dependant manner, is correlated with CRC metastasis (46,47). Increased cell surface expression of LAMP1, a lysosomal membrane glycoprotein expressed in the lysosome and at the cell surface, is associated with the metastatic potential of CRC (48). Also, this protein is a ligand of E-selectin and P-selectin of endothelial cells, and its increased expression facilitates the attachment of cancer cells to activated endothelial cells (49). LCK, a member of the Src family, plays an essential role in the selection and maturation of developing lymphoid cells. Moreover, metastatic cancers express high levels of LCK compared to their primary cancer (50). LCP1 expression is significantly correlated with the progression of CRC staging (51). Expression of MACC1 is significantly upregulated in CRC compared to normal tissues and correlated with low survival rates inducing migration, invasion and proliferation of cancer cells (52). SLC12A2 is overexpressed in CRC (53) and liver metastatic CRC (54). TAGLN2 is a member of the calponin family of actin-binding proteins potentially involved in cytoskeletal organization. Its overexpression is observed in lymph nodes and other distant metastatic regions of the CRC and associated with a reduced survival rate (24). In addition, the overexpression of VIM represents a higher malignant potential of CRC considering as a useful biomarker for survival in CRC patients (55).

Moreover, AK2 (56), HSPA9 (57) and LGALS3BP (58) are overexpressed in metastatic cancer cells. S100 proteins are commonly upregulated in various cancers including breast, bladder, colorectal, gastric, kidney, lung, prostate and thyroid cancers (59). They are low-molecular-weight calcium-binding family proteins specifically expressed according to cell type and environmental condition. Moreover, increased expression of S100A4, S100A6 and S100A9 may be associated with CRC invasion and metastasis (59,60), and S100A4 and S100A14 expression correlates with metastatic potential and prognosis in CRC (61). SDCBP and SDCBP2 are PDZ-domain-containing scaffold proteins involved in a variety of cellular processes. Several studies have suggested that SDCBP plays a role in cancer metastasis by increasing migration through modulation of FAK activity (62).

Some upregulated proteins in SW620 EVs are related to multidrug resistance such as ABCC4, CACYBP and GSTP1. Chemotherapeutics are the most effective treatment for metastatic cancer but cancer cells can acquire simultaneous resistance to different drugs, a phenomenon known as multidrug resistance (63). Multidrug transporter proteins contribute to chemoresistance via the efflux of anticancer drugs from host cancer cells. We found ABCC4 and ABCC1 were 1.61-fold and 1.30-fold upregulated, respectively, in SW620 EVs compared to SW480 EVs. Overexpression of ABCC1 and ABCC4 are poor prognostic indicators in neuroblastoma and predictive markers of adverse clinical outcomes (64). Importantly, these multidrug transporter proteins may be transferred to other cancer cells via EVs (65). Overexpression of CACYBP, which interacts with S100A6 (calcyclin), enhances gastric cancer resistance to multiple chemotherapeutic drugs (66). Also, comparative proteomic analysis of SW480 and SW620 cells shows that CACYBP is overexpressed in SW620 cells (12). CACYBP modulates adhesion characteristics via reduction of cellular CTNNB1 expression (12). GSTP1, a member of the glutathione S-transferase enzyme, is commonly overexpressed in CRC, and its increased expression is associated with a poor clinical prognosis and multidrug resistance (67). However the involvement of these proteins in the metastatic process has not been evaluated. Thus, further investigations will provide valuable information on the role of EVs in the metastatic process.

## Conclusion

EVs may have clinical applications as diagnostic tools or vaccines against cancers, as well as play roles in intercellular communication under pathophysiological conditions (1,21). In this study, we conducted the first quantitative proteomics comparison between primary and metastatic cancer cell-derived EVs. Our results show that these EV proteomes are enriched with plasma membrane proteins and distinguishable from the secretomes (which were enriched with extracellular proteins). Based on quantitative proteomic approach, we found that cell adhesion related proteins are enriched in EVs derived from primary cancer, SW480. In contrast, SW620 EV-enriched proteins are involved in cancer progression and function as diagnostic indicators of metastatic cancer with poor prognosis; they are overexpressed in CRC/metastatic cancer and play roles in multidrug resistance. Although our comparative proteomics does not give a complete picture of the roles of EVs in the metastatic process, our proteomic data provide valuable information toward future research on the pathological function of EVs in metastasis. Furthermore, this study presents useful biomarkers to develop EV-based diagnostics for metastatic CRC.
